# Bioinformatics Analysis of Genes Associated with Autophagy and Metabolic Reprogramming in Atrial Fibrillation

**DOI:** 10.3390/jcdd13020082

**Published:** 2026-02-08

**Authors:** Yaqianqian Niu, Kensuke Ihara, Satoshi Iwamiya, Tetsuo Sasano

**Affiliations:** Department of Cardiovascular Medicine, Institute of Science Tokyo, 1-5-45 Yushima, Bunkyo-ku, Tokyo 113-8519, Japan; yaqianqian.cvm@tmd.ac.jp (Y.N.); satoshi-iwamiya.cvm@tmd.ac.jp (S.I.); sasano.cvm@tmd.ac.jp (T.S.)

**Keywords:** atrial fibrillation, autophagy, metabolic reprogramming, key gene

## Abstract

Atrial fibrillation (AF) is the most common cardiac arrhythmia, and both metabolic reprogramming and autophagy have been implicated in its pathogenesis. However, the expression pattern of autophagy-related genes during metabolic reprogramming in AF remains elusive. We aimed to characterize the expression profiles of autophagy- and metabolic reprogramming-related genes in atrial tissue to gain pathophysiological insights into AF. Three datasets obtained from the Gene Expression Omnibus (GSE2240, GSE79768, and GSE14975) that included atrial tissue samples from patients with or without AF were subjected to a bioinformatics analysis, which identified 2812 differentially expressed genes. Eight autophagy- and metabolic reprogramming-related differentially expressed genes (A&MRRDEGs) were identified as key candidates through least absolute shrinkage and selection operator regression combined with the random forest approach. Meanwhile, mice underwent transverse aortic constriction (TAC) for 2 weeks in an AF model, and gene expression in atrial tissue was analyzed. In atrial tissues from TAC mice, only *Akt1* and *Hspa5* of the eight A&MRRDEGs exhibited expression changes concordant with the human datasets, while *Glud1* showed discordant regulation. Collectively, these cross-species findings highlight that the eight A&MRRDEGs, particularly *AKT1* and *HSPA5*, are potentially involved in autophagy and metabolic reprogramming during AF pathogenesis.

## 1. Introduction

Atrial fibrillation (AF) is the most prevalent form of cardiac arrhythmia, and its occurrence drives a heightened risk of stroke, long-term disability, and mortality [1,2]. Existing pharmacological treatments for AF remain suboptimal in preventing the condition from exacerbating and may induce significant proarrhythmic effects [3]. The limited effectiveness of current antiarrhythmic medications is likely attributable to a knowledge gap regarding the mechanisms that initiate and maintain AF [4]. Therefore, there is an urgent need to elucidate novel pathophysiological mechanisms, which will provide the basis for developing new diagnostic and therapeutic strategies aimed at improving clinical outcomes.

Metabolic disorders, including diabetes mellitus and obesity, increase the risk of AF [2]. However, current therapeutic approaches for these conditions often fail to prevent the development of AF [3]. During episodes of AF, rapid atrial excitation leads to incomplete and irregular atrial contractions, which in turn increases energy requirements and necessitates subsequent adaptations in the metabolic supply [5]. Although metabolic reprogramming initially serves as a physiological response to satisfy the high atrial energy demands, it can evolve into a chronic maladaptive process that promotes the formation of an arrhythmogenic substrate conducive to AF [5]. Focusing on atrial metabolism reprogramming thus represents a promising therapeutic strategy that directly addresses the metabolic stress and reprogramming associated with AF. Metabolic and energetic stress can also disrupt autophagy—a crucial catabolic metabolic mechanism in eukaryotic cells, facilitating efficient degradation of various macromolecules [6]. Autophagy is involved in maintaining intracellular homeostasis, primarily by reducing reactive oxygen species through the clearance of dysfunctional mitochondria [7]. Autophagy dysfunction is a dysregulated metabolic process implicated in numerous diseases, including AF [8]. In AF, autophagy is associated with electrical remodeling, anatomical changes, neural remodeling, and, importantly, alterations in energy metabolism [9]. Analyses of differential gene expression, functional enrichment, and protein–protein interactions concerning autophagy in AF have indicated that autophagy-related genes (ARGs) could function as indicators and therapeutic focal points for AF [10]. A recent comprehensive study examining ARGs in relation to valvular AF underscored their potential utility as predictive markers and therapeutic targets for guiding treatment strategies in patients with AF [11].

However, the precise expression patterns of ARGs during metabolic reprogramming in AF remain elusive. Thus, we examined the expression patterns of autophagy- and metabolic reprogramming-related differentially expressed genes (A&MRRDEGs) in AF to identify candidate key genes related to autophagy and metabolic reprograming in AF pathogenesis.

## 2. Materials and Methods

The main bioinformatics analysis workflow used in this study was established based on previously published studies [12,13,14,15]. The procedures are detailed in the following sections.

### 2.1. Study Datasets

Using the R package GEOquery [16] (version 2.70.0), we retrieved three human-derived transcriptome datasets from the Gene Expression Omnibus (GEO) database [17]. The research subjects were humans and the atrial tissue was used for the study. Furthermore, a microarray detection platform was adopted. According to the inclusion criteria, the dataset should simultaneously include the AF and sinus rhythm (SR) groups. The sample size of each group should be no less than five cases, and only cases of non-valvular AF were allowed. Ultimately, only three datasets, namely GSE2240, GSE79768 and GSE14975, met the above criteria. The detailed information of the datasets adopted in this study is shown in Table S1.

To identify ARGs, we searched the GeneCards database [18] (https://www.genecards.org/, accessed on 6 May 2024) using the keyword “Autophagy” and filtered for “Protein Coding” genes, yielding 7327 ARGs. An additional 23 ARGs were identified through a PubMed literature search using the same keyword [19]. Finally, after consolidating both sources and removing duplicates, 7350 ARGs were included in the dataset (Datasheet S1).

We sourced the initial gene set for metabolic reprogramming-related genes (MRRGs) from the GeneCards resource [14] by using “Metabolic Reprogramming” as the keyword and filtering for key genes, yielding 1423 MRRGs. A complementary PubMed search using the same keyword identified five additional MRRGs from the literature [20]. Merging both sources yielded a total of 1424 MRRGs (Datasheet S2). Eventually, we combined the ARGs and MRRGs to construct a dataset containing 1137 autophagy- & MRRGs (A&MRRGs), as detailed in Datasheet S3.

### 2.2. AF-Related A&MRRDEGs

The R package sva [21] (version 3.50.0) was used to correct for batch effects across the GSE2240, GSE79768, and GSE14975 datasets, resulting in an integrated GEO dataset (combined datasets) comprising 29 AF and 37 SR cases. The R package limma (Version 3.58.1) [22] was subsequently employed to normalize and standardize the combined datasets. Principal component analysis [23] was performed on the expression matrix before and after batch effect correction to evaluate the effectiveness of batch effect removal.

After examining the combined datasets, we categorized the samples into the AF and SR groups. The R package limma was used for differential gene expression analysis, and genes with log fold change |logFC| > 0 and *p*-value < 0.05 were considered as differentially expressed genes (DEGs). Genes exhibiting a logFC > 0 and a *p*-value < 0.05 were classified as upregulated DEGs, while genes with a logFC < 0 and a *p*-value < 0.05 were recognized as downregulated DEGs. The Benjamini–Hochberg (BH) method was applied to correct the *p*-values. We employed the R package ggplot2 (version 3.4.4) to create a volcano plot for visually representing the outcomes of the differential gene expression analysis.

To identify A&MRR-related DEGs associated with AF, all DEGs meeting the |logFC| > 0 and *p*-value < 0.05 criteria from the combined dataset were integrated with A&MRRGs. Subsequently, the A&MRRDEGs were extracted and a Venn diagram was constructed to show the overlaps. Additionally, a heat map depicting the top 20 A&MRRDEGs was created using the R package pheatmap (version 1.0.12).

### 2.3. Gene Ontology (GO) and Pathway Enrichment Analysis

We utilized the R package Cluster Profiler (version 4.10.0) [24] to show the results of the GO analysis [25] and pathway enrichment analyses of A&MRRGs. The selection criteria for items were determined with a significance level set at *p* < 0.05, and a false discovery rate (FDR) threshold (q-value) of <0.05. This was achieved by utilizing the BH procedure for the correction of *p*-values. Subsequently, the R package pathview (version 1.42.0) [26] was employed to visualize the Kyoto Encyclopedia of Genes and Genomes (KEGG) [27] analysis through related circuit diagrams.

### 2.4. Gene Set Enrichment Analysis (GSEA) and Gene Set Variation Analysis (GSVA)

GSEA [28] evaluates the contribution of genes to the observed phenotype. In this study, the genes from the combined datasets were initially sequenced according to the logFC values, distinguishing the AF from the SR group. Subsequently, the R package Cluster Profiler (version 4.10.0) [24] was used to perform GSEA across all genes in the combined datasets. The parameters included a random seed of 2020, with each gene set comprising a maximum and minimum of 500 and 10 genes, respectively. Access to the c2.all.v2023.2.Hs.symbols gene sets was granted via the Molecular Signatures Database (MSigDB). The criteria for inclusion in the enrichment analysis were a *p*-value < 0.05 and FDR (q-value) < 0.05. The *p*-value was adjusted using the BH procedure.

GSVA transforms gene expression matrices between samples into corresponding gene set expression matrices, thereby assessing whether distinct biological pathways exhibit enrichment across various samples [29]. Access to the c2 gene sets was again facilitated through the MSigDB [30], specifically Cp.V2023.2.Hs.Symbols. To evaluate functional enrichment differences between the AF and SR groups, we applied GSVA to all genes in the combined GEO datasets using the R package GSVA (version 1.50.0). Statistical significance was set at *p* < 0.05, with multiple testing corrected using the BH method.

### 2.5. Construction of the AF Diagnostic Model

To derive the diagnostic models for AF using the combined GEO datasets, logistic regression analysis was conducted on the A&MRRDEGs. This analysis treated the dependent variable as binary, distinguishing between the AF and SR groups, thereby assessing the relationship between the independent and dependent variables. A significance threshold of *p* < 0.05 was established for the selection of A&MRRDEGs to construct the logistic regression model.

Subsequently, least absolute shrinkage and selection operator (LASSO) analysis was performed on these genes using the R package glmnet (Version 4.1-10) [31], with parameters set as set.seed (500) and family = “binomial” This approach builds on linear regression by introducing a penalty term—the product of the absolute values of lambda and slope—to reduce overfitting and improve model generalizability. The resulting diagnostic model for AF incorporated A&MRRDEGs as predictor variables. the LASSO risk score was generated using the defined risk coefficients, following the specified calculation formula in Equation (1).

(1) $$riskScore=\sum_{i}Coefficient\;\left({gene}_{i}\right)*mRNA\;Expression\;({gene}_{i})$$

The random forest (RF) [32] technique is widely recognized as an effective approach for model construction. When a prediction is required for a specific sample, the algorithm collects the predictive outputs from each tree within the ensemble and subsequently derives the final prediction through a voting mechanism. In our analysis, we utilized the RF package to construct models predicated on the expression levels of A&MRRDEGs. The parameters were established using set.seed (234) and ntree = 200.

We implemented a 5-fold cross-validation process, conducting 10 repetitions to refine the selection of variables. Cross-validation on the training dataset was used to identify variables with lower error rates, and the most informative features were subsequently prioritized according to their mean decrease Gini scores. Genes identified from the intersection of A&MRRDEGs, as determined by both LASSO regression and RF analyses, were designated as key genes (mRNA) for subsequent analyses.

### 2.6. Validation of AF Diagnostic Model

We employed the R package rms (Version 8.0-0) to build a nomogram for the key genes. To assess the predictive efficacy of our model against the actual outcomes, we generated a calibration plot. We conducted a decision curve analysis (DCA) using the R package ggDCA (Version 1.1)to evaluate the clinical net benefit and practical decision-making value of the logistic regression model.

Moreover, we utilized the R package pROC (Version 1.18.5) to generate receiver operating characteristic (ROC) curve within the combined datasets and calculate the area under the curve (AUC). This analysis aids in evaluating the diagnostic capability of the logistic regression models concerning the incidence of AF.

### 2.7. Construction of Regulatory Network

Micro RNAs (miRNAs) are crucial for modulating developmental and evolutionary processes. They have the capacity to influence a diverse array of target genes, and notably, a single gene may be regulated by multiple miRNAs. To identify miRNAs targeting A&MRRDEGs, we utilized the TarBase (http://www.microrna.gr/tarbase, accessed on 6 May 2024) [33] and StarBase V3.0 database (https://starbase.sysu.edu.cn/, accessed on 6 May 2024) [34] and visualized the results using the Cytoscape software (version3.10.1).

Transcription factors (TFs) are involved in regulating gene expression through their interactions with A&MRRDEGs. Using the ChIPBase [35] database (http://rna.sysu.edu.cn/chipbase/, accessed on 6 May 2024), we retrieved TFs and explored their regulatory associations with A&MRRDEGs. The resulting mRNA–TF regulatory network was visualized using the Cytoscape software [36].

### 2.8. Expression Patterns of Key Genes with Variance Analysis

To elucidate the potential mechanistic role of A&MRRDEGs in AF alongside their characteristics, we employed the Mann–Whitney U test to measure the differences in key genes between the AF and SR groups. The findings were subsequently visualized in a comparative grouping chart using the R package ggplot2 (version 3.4.4).

Subsequently, we identified the key genes and further examined their ROC curves [37] within the combined datasets. AUC values between 0.5 (including 0.5) and 0.7 suggest low accuracy, values between 0.7 (including 0.7) and 0.9 imply moderate accuracy, and values of 0.9 and above indicate high accuracy. Utilizing the R proc package, we constructed the ROC curve for the identified key genes and calculated the AUC to evaluate the diagnostic implications of A&MRRDEGs expression for patients diagnosed with AF.

### 2.9. Immune Infiltration Analysis [Using Single-Sample (ssGSEA)]

A large body of evidence indicates that immune and inflammatory responses play significant roles in the occurrence and development of AF [6]. Meanwhile, autophagy and metabolic reprogramming are closely related to the function of immune cells [38]. The enrichment fractions derived from ssGSEA [39] were utilized to reflect the abundance of immune cell infiltration in each sample (expressed as a matrix). The R package ggplot2 (version 3.4.4) was used to visualize the comparison of immune cell distributions. Differences between the two groups were identified for further investigation.

Spearman correlation analysis was performed to assess the correlations among immune cells, and the R-PHEAT map (version 1.0.12) was used to generate a heatmap illustrating the interrelationships among the immune cells. Additionally, the Spearman correlation analysis was also used to analyze the association between the key genes and immune cells, described by means of a bubble map generated using the ggplot2 package (version 3.4.4).

### 2.10. Feature Similarity Analysis

The GOSemSim package (Version 2.34.0) was used to calculate the semantic similarity pertaining to key genes. Subsequently, the geometric mean of the key genes was determined across the biological process, cellular component, and molecular function categories to derive the ultimate scores.

### 2.11. Mice and Transverse Aortic Constriction (TAC) Operation

All animal research was performed in accordance with the Guidelines for the Care and Use of Laboratory Animals, published by the National Research Council (The National Academy Press, 8th edition, 2011). The study protocol was approved by the Institutional Animal Care and Use Committee of the Institute of Science Tokyo (Approval #A2024-103). Male wild-type mice (C57BL/6jjcl) were purchased from CLEA Japan, Inc., Tokyo, Japan. All procedures were performed at the animal laboratory (#2030-005) of Institute of Science Tokyo. Mice were acclimatized for at least 1 week before surgery. Mice were housed under conventional conditions with controlled temperature (22 ± 2 °C) and humidity (40–60%) under a 12-h light/dark cycle, with ad libitum access to standard chow and water. In accordance with previously established protocols [40], C57BL/6jjcl mice aged between 9 and 11 weeks (weight: 20–25 g; total *n* = 12) underwent either TAC (*n* = 6) or sham surgery (*n* = 6, control group) in a random manner. The experimental unit was the individual mouse. TAC was performed using 7-0 polypropylene sutures (Ethicon, Inc., Somerville, NJ, USA) with a 27-gauge needle (Terumo Corporation, Tokyo, Japan) serving as a guide, under 1.5% isoflurane anesthesia [41]. Animals were kept on a warming pad during and after surgery and were monitored until full recovery. Humane endpoints were predefined. Mice were euthanized if respiratory distress was observed during the experimental period.

### 2.12. In Vivo Electrophysiological Study

Mice were subjected to surface ECG under anesthesia (1.5% isoflurane inhalation) in a blinded manner on postoperative day 14, employing a lead II configuration [42,43].

To induce AF [41], transesophageal electrical stimulation was performed. A custom-made bipolar electrode catheter (Unique Medical, Tokyo, Japan), intended for pacing purposes, was inserted into the posterior atrium through the esophagus while the animal was anesthetized (1.5% isoflurane). Proper positioning of the catheter along the dorsal side of the left atrium was confirmed by observing complete P-wave capture, with consistent appearance between QRS complexes. The minimum capture threshold was established at this stage. Twice the threshold voltage was delivered during subsequent programmed electrical stimulations. Atrial stimulation was conducted using the PowerLab system (ADInstruments, Dunedin, New Zealand), which included baseline pacing (S1) and up to three extra stimuli (S2–S4). S1 consisted of 10 atrial stimuli at a pacing cycle length of 80 ms. S2 was delivered 40 ms after the last S1 stimulus, with coupling intervals progressively shortened in 5-ms steps until the effective refractory period (ERP) was reached. S3 was introduced 40 ms after S2 and similarly decremented in 5-ms steps until ERP. S4 was applied after S3 using the same protocol [41].

AF was diagnosed based on the episodes of irregular RR intervals with concomitant absence of P-waves that persisted for >1 s [44]. AF duration was defined as the period beginning with the onset of arrhythmic atrial contractions and concluding with the initiation of the first beat of restored SR. Throughout the procedure, body temperature was maintained at 37 °C with a light bulb.

### 2.13. In Vivo Echocardiogram Study

Transthoracic echocardiography was conducted utilizing a Vinno 6LAB ultrasound diagnostic device (VINNO Technology, Suzhou, China) on day 14 following either TAC or sham surgery, with the procedure being performed in a blinded manner. The heating pad of the device and a light bulb were used to maintain the body temperature of the anesthetized mice (1.5% isoflurane) at 37 °C. Various cardiac parameters were assessed in the long-axis view through 2D-guided M-mode imaging, including left atrial diameter (LAD), systolic and diastolic interventricular septal thickness (IVST, systolic/diastolic), systolic and diastolic left ventricular posterior wall thickness (LVPWT, systolic/diastolic), left ventricular end-diastolic diameter (LVEDD), left ventricular end-systolic diameter (LVESD), ejection fraction (EF), and fractional shortening (FS). The reported averages were derived from a minimum of three measurements.

### 2.14. Isolation of Atrium and Quantitative Real-Time Polymerase Chain Reaction (qPCR)

Cervical dislocation was employed to sacrifice TAC and sham mice (*n* = 6 each). The hearts were swiftly excised, and the left atrium was separated from the entire murine heart. Total RNA was extracted from the left atria of both TAC and sham mice using the RNeasy Mini Kit (QIAGEN, Hilden, Germany) in accordance with the manufacturer’s instructions. Total RNA (200 ng) derived from the atrial tissue was used to synthesize cDNA using a High-Capacity cDNA Reverse Transcription Kit (Thermo Fisher Scientific, Waltham, MA, USA). To assess the mRNA levels of key genes, qPCR was performed using THUNDERBIRD qPCR reagents (TOYOBO, Osaka, Japan) according to the manufacturer’s instructions and primers synthesized by Fasmac. The primer sequences are provided in Table S2. Expression levels were normalized to those of *Gapdh*. Data analysis was conducted using the comparative 2^−ΔΔCT^ method to determine relative gene expression.

### 2.15. Statistical Analysis

All data processing procedures and analyses presented in this study were conducted using R software (version 4.2.2). To assess the statistical significance of continuous variables between two distinct groups, normally distributed variables were evaluated using the independent Student *t*-test, unless indicated otherwise. In instances where the data did not follow a normal distribution, the Mann–Whitney U test was employed to analyze the differences among the variables. Spearman’s correlation analysis was performed to determine the correlation coefficients between various molecules. The Fisher’s exact test was employed to evaluate the statistical significance of AF inducibility. Unless stated otherwise, all statistical *p*-values were two-sided with a significance threshold of *p* < 0.05.

## 3. Results

### 3.1. Discovery of 265 A&MRRDEGs in AF

**Figure 1:**
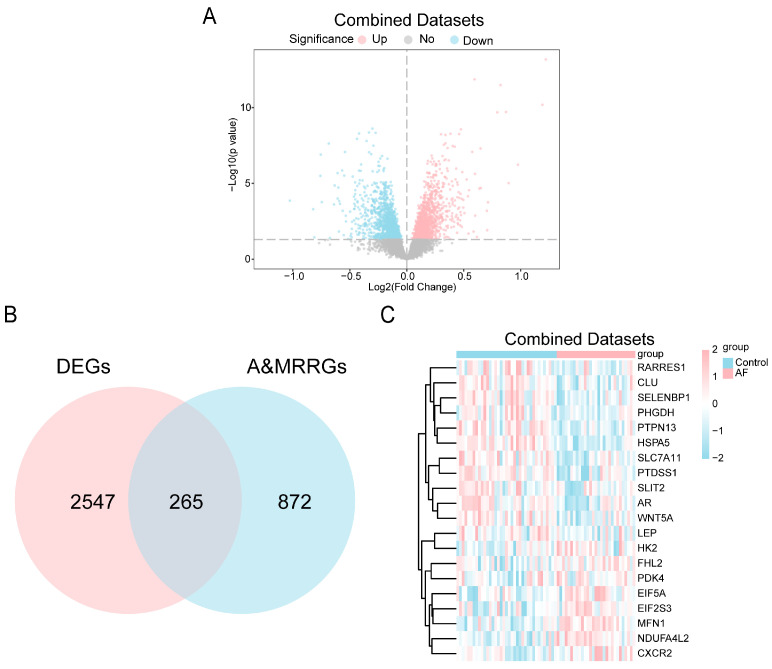
Differential Gene Expression Analysis. (**A**) Volcano plot of differentially expressed genes between AF and SR groups in the combined GEO datasets. Upregulated genes are shown in pink, down-regulated genes in blue, and non-significantly different genes in gray. The horizontal dotted line indicates the *p*-value threshold (0.05). (**B**) Venn diagram of combined datasets of DEGs and A&MRRGs. (**C**) Heat map of the top 20 A&MRRDEGs in the combined datasets; pink represents high expression and blue represents low expression. Abbreviations: AF, Atrial fibrillation; A&MRRGs, Autophagy- & metabolic reprogramming-related genes; DEGs, Differentially expressed genes.

To investigate alterations of A&MRRGs in AF, we analyzed public datasets, GSE2240 [45], GSE79768 [46], and GSE14975 [47], containing gene expression data from human atrial tissue. After batch effect correction, expression values were homogenized across datasets, and subsequent principal component analysis confirmed that inter-batch variability was effectively eliminated (Figure S1). Differential expression analysis identified 2812 DEGs, including 1434 upregulated and 1378 downregulated genes (Figure 1A). By intersecting these DEGs with A&MRRGs curated from online databases, 265 A&MRRDEGs were obtained, including *PGM1*, *HSPA5*, *AKT1*, *ARPC4*, *VDAC1*, *GLUD1*, *YWHAQ*, *TOMM22*, *P4HA1*, *SLC25A5*, *MFN1*, *NDUFA4L2*, *RTN4*, and *PDAC1* (Figure 1B,C).

### 3.2. Functional Enrichment Analysis

**Figure 2:**
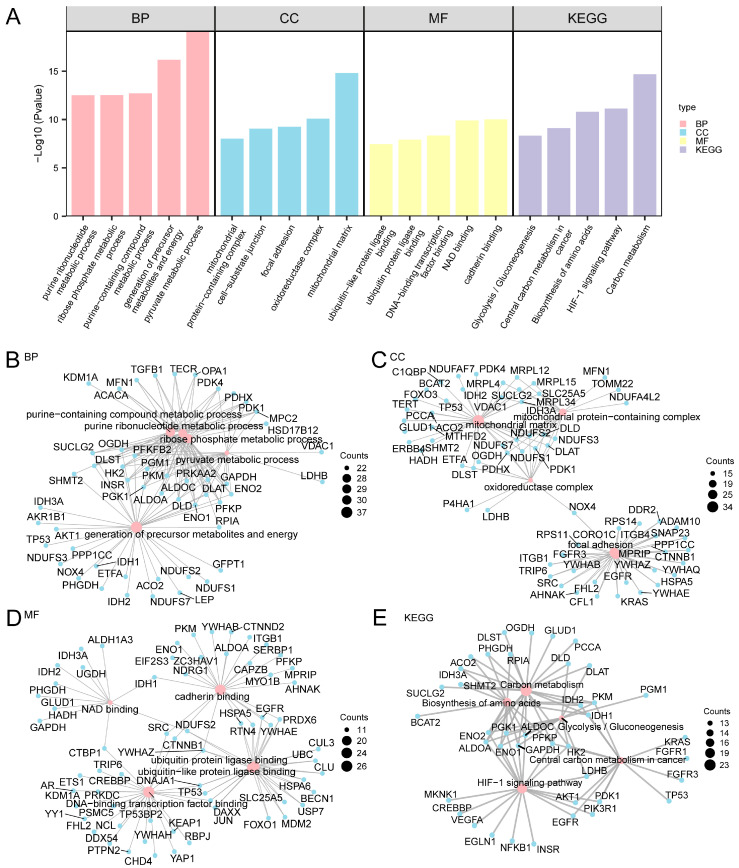
GO and KEGG Enrichment Analysis for A&MRRDEGs. (**A**) Histogram showing the results of GO and KEGG pathway enrichment analyses for A&MRRDEGs, categorized into BP, CC, MF, and KEGG pathways. The *x*-axis displays the enriched GO and KEGG terms. (**B**–**E**) Network diagrams illustrating GO and KEGG enrichment results for A&MRRDEGs: BP (**B**), CC (**C**), MF (**D**), and KEGG pathways (**E**). Pink nodes represent enriched terms, while blue nodes indicate associated genes; edges represent gene-term associations. The enrichment analyses were performed using *p* < 0.05 and FDR or q-value < 0.05 as thresholds. Multiple testing correction was applied using the BH method. Abbreviations: BP, Biological process; CC, Cellular component; FDR, False discovery rate; GO, Gene ontology; KEGG, Kyoto Encyclopedia of Genes and Genomes; MF, Molecular function.

To gain more biological insight and investigating the crucial pathways, by GO enrichment analysis, the 265 A&MRRDEGs in AF were further explored (Figure 2A and Table S3).

Functional enrichment analysis indicated that the 265 A&MRRDEGs were predominantly associated with processes such as precursor metabolite and energy generation, purine-containing compound metabolism, and ribose phosphate metabolic pathways. At the molecular function level, significant enrichment was observed in DNA-binding transcription factor interactions as well as in ubiquitin and ubiquitin-like protein ligase binding activities.

KEGG pathway analysis further revealed significant enrichment in carbon metabolism, amino acid biosynthesis, glycolysis, gluconeogenesis, and related metabolic pathways. A network diagram was generated to visualize the interrelationships among enriched GO terms of BP, CC, MF, and KEGG pathways (Figure 2B–E).

### 3.3. GSEA and GSVA

We adopted GSEA and GSVA in our research to systematically and comprehensively reveal the abnormal states of related molecular mechanisms and functional pathways during AF from the perspectives of the overall enrichment trend of the gene set and the changes in pathway activity at the sample level.

**Figure 3:**
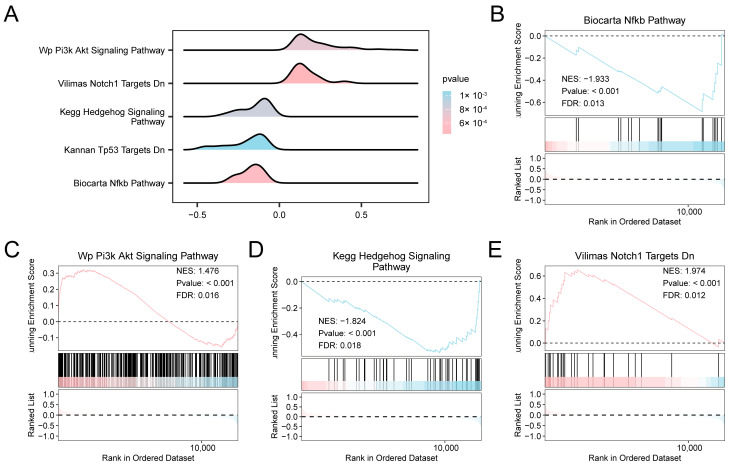
Differential Gene Expression Analysis and GSEA for combined datasets. (**A**) GSEA of the combined GEO datasets, showing enrichment plots (“mountain plots”) for five significantly enriched biological functions. (**B**–**E**) GSEA results indicating significant enrichment of specific pathways in the combined datasets: Biocarta NF-κB Pathway (**B**), WP PI3K-Akt Signaling Pathway (**C**), KEGG Hedgehog Signaling Pathway (**D**), and Vilimas Notch1 Targets Downregulated (**E**). The color gradient of the enrichment plots reflects the significance of the enrichment: pink shades indicate lower *p*-values, while blue shades indicate higher *p*-values. GSEA was performed using a significance threshold of *p* < 0.05 and FDR (q-value) < 0.05, with multiple testing correction performed using the BH method. Abbreviations: FDR, False discovery rate; GEO, Gene Expression Omnibus; GSEA, Gene set enrichment analysis; KEGG, Kyoto Encyclopedia of Genes and Genomes.

We investigated the relationship between the expression profiles of all genes in the combined datasets to evaluate the influence of gene expression levels on the development of AF. GSEA revealed significant enrichment of multiple signaling cascades, such as NF-κB, PI3K–Akt, Hedgehog, and Notch Targets (Figure 3A–E, Table S4). To further explore pathway-level differences between AF and SR, GSVA was performed. GSVA identified multiple pathways significantly altered in AF, particularly those related to apoptotic signaling, HIF-1 regulation, MAPK/ERK signaling, mitochondrial electron transport, and lipid metabolism (Figures S2 and S3; Table S5).

### 3.4. Eight Key A&MRRDEGs

**Figure 4:**
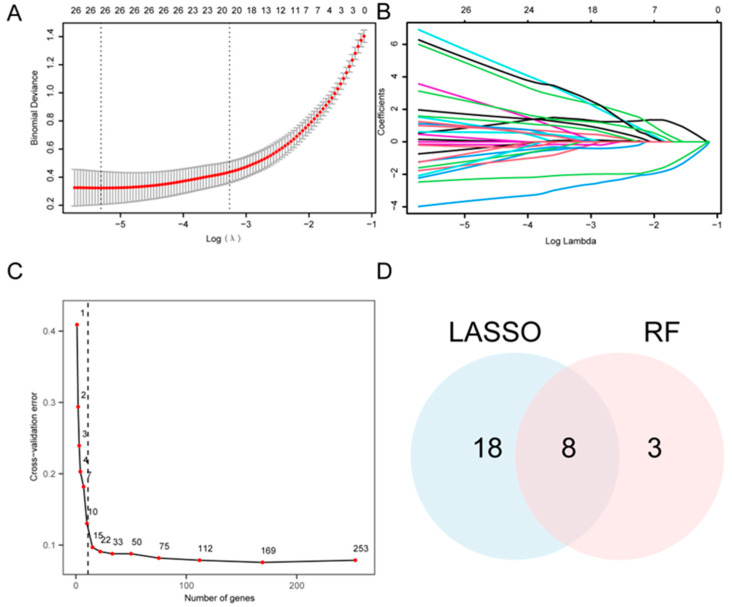
Construction of the Diagnostic Model for AF. (**A**) LASSO regression model based on A&MRRDEGs identified from the combined GEO datasets. Red dots show the binomial deviance at each value of the regularization parameter λ, and gray vertical bars indicate standard error. The vertical dotted lines denote λ that minimizes the deviance and the largest λ within 1 standard error of the minimum. Numbers along the top indicate the number of genes included in the model at each λ. (**B**) Trajectory plot of variables in the LASSO diagnostic model shows the changes in coefficients of different genes at various λ values. (**C**) Cross-validation error curve used to determine the optimal number of genes for model construction. The red dot indicates the number of genes selected in the optimal model. The vertical dotted line indicates the selected optimal model (11 genes). (**D**) Venn diagram showing the overlap between gene selections from the LASSO regression and RF algorithms. Abbreviations: AF, Atrial fibrillation; A&MRRDEGs, Autophagy- and metabolic reprogramming-related differentially expressed genes; LASSO, Least absolute shrinkage and selection operator; RF, Random forest.

Univariate logistic regression analysis was performed to evaluate the diagnostic significance of the 265 A&MRRDEGs in AF, which revealed 253 genes with statistically significant association (Datasheet S4). Accordingly, a LASSO regression model was developed and yielded 26 candidate genes (Figure 4A,B). In parallel, a RF algorithm was applied, and feature selection identified 11 genes with the highest relevance to AF diagnosis (Figure 4C). Intersection analysis between the LASSO- and RF-derived gene sets revealed eight key A&MRRDEGs: *AKT1*, *ARPC4*, *GLUD1*, *HSPA5*, *NSDHL*, *PGM1*, *PIN1*, and *VDAC1* (Figure 4D).

### 3.5. Validation of the Diagnostic Model

**Figure 5:**
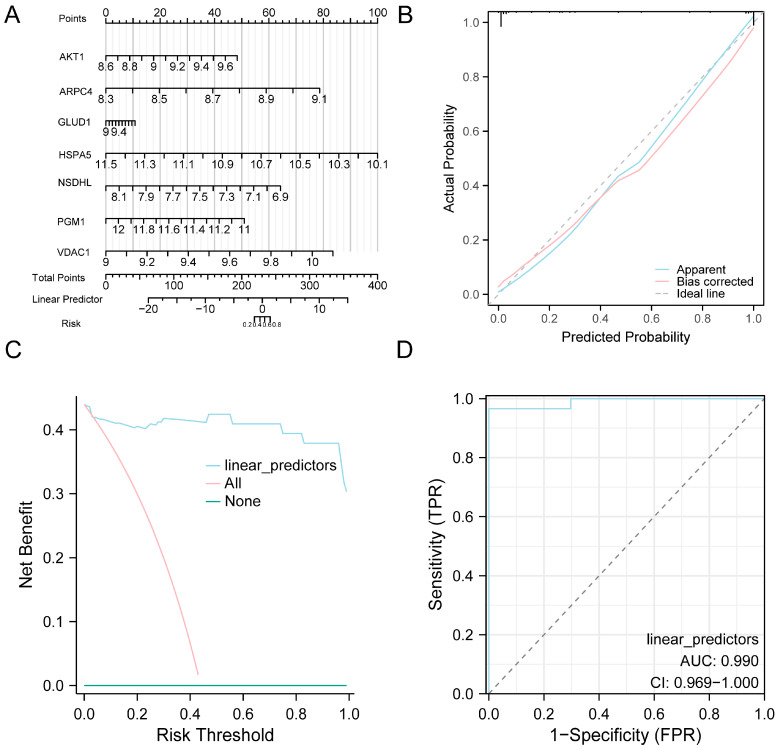
Diagnostic and Validation Analysis of AF. (**A**) Nomogram illustrating the contribution of model genes to the AF diagnostic model constructed from the combined GEO datasets. (**B**,**C**) Validation of the AF diagnostic model based on the combined GEO datasets using calibration curve analysis (**B**) and DCA (**C**). (**D**) ROC curve of the logistic regression model in the combined GEO datasets, based on the linear predictors derived from the model. In the DCA plot, the *y*-axis represents net benefit, while the *x*-axis indicates the threshold probability. Abbreviations: AF, Atrial fibrillation; AUC, Area under the curve; DCA, Decision curve analysis; GEO, Gene Expression Omnibus; ROC, Receiver operating characteristic.

To validate the diagnostic utility of the eight-gene model for AF, a nomogram was generated wherein *HSPA5* exhibited the strongest contribution to AF prediction (Figure 5A). Calibration analysis showed that the predicted probabilities were generally consistent with the observed outcomes, with only slight deviation from the ideal line (Figure 5B). DCA demonstrated that the proposed model achieved higher net clinical benefit than either the treat-all or treat-none approaches (Figure 5C). Furthermore, ROC curve analysis demonstrated good discriminative performance of the model in distinguishing AF from SR in the combined datasets (Figure 5D).

### 3.6. Construction of Regulatory Networks

MiRNAs predicted to target the A&MRRDEGs, as identified using the TarBase and StarBase V3.0 databases, formed the basis for the mRNA–miRNA interaction map, which is shown in Figure S3A. This network comprised 8 A&MRRDEGs and 138 miRNAs (Datasheet S5).

Then, the TFs combined with A&MRRDEGs were taken advantage to build the mRNA–TF regulatory network and the result was visualized in Figure S3B. Among them, there were 8 A&MRRDEGs and 76 TFs (Datasheet S6).

### 3.7. Expression and Diagnostic Performance of Key Genes

**Figure 6:**
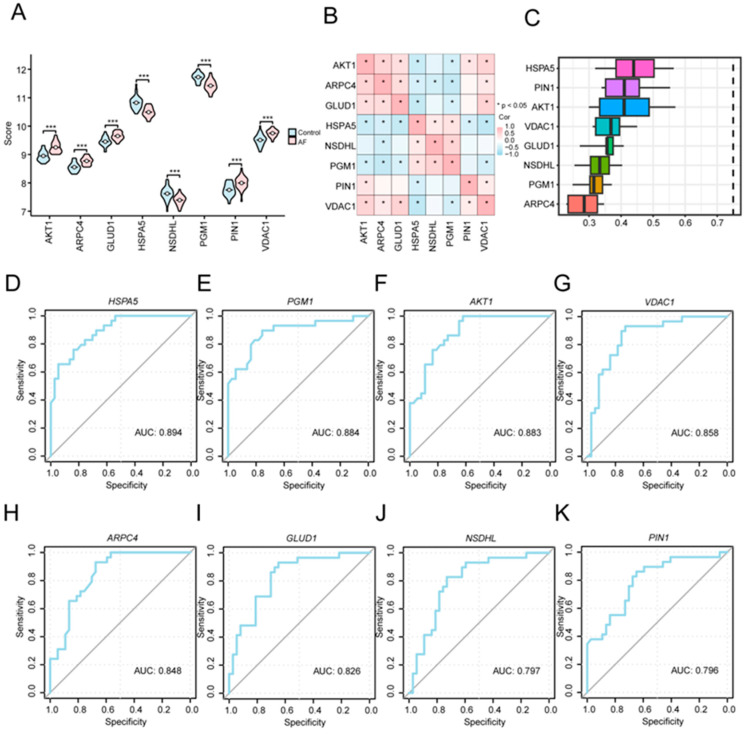
Expression Differences and Diagnostic Performance of Key Genes between AF and SR Groups. (**A**) Comparison of key gene expression levels between the AF and SR groups in the combined GEO datasets. (**B**) Correlation analysis among the key genes. (**C**) Functional similarity analysis of the key genes. (**D**–**K**) ROC curve analyses of key genes: *HSPA5* (**D**), *PGM1* (**E**), *AKT1* (**F**), *VDAC1* (**G**), *ARPC4* (**H**), *GLUD1* (**I**), *NSDHL* (**J**), and *PIN1* (**K**) in the combined GEO datasets. Asterisks (*** *p* < 0.001) indicate high statistical significance. Abbreviations: AF, Atrial fibrillation; AUC, Area under the curve; GEO, gene expression omnibus; ROC, Receiver operating characteristic.

For further evaluating the biological and diagnostic relevance of the eight key A&MRRDEGs, we compared their expression profiles between the AF and SR groups and examined their interrelationships. These eight genes (*AKT1*, *ARPC4*, *GLUD1*, *HSPA5*, *NSDHL*, *PGM1*, *PIN1*, and *VDAC1*) showed significantly different expression levels between the two groups (*p* < 0.001, Figure 6A). Correlation analysis revealed positive associations among *ARPC4*, *AKT1*, *VDAC1*, and *GLUD1*, whereas *HSPA5* was negatively correlated with *AKT1*, *GLUD1*, and *VDAC1* (Figure 6B). Functional similarity analysis further indicated that these genes share substantial overlap in GO annotations, with *HSPA5* demonstrating the highest similarity to the others (Figure 6C). ROC curve analysis confirmed that each of the eight key genes individually possessed diagnostic value for AF (Figure 6D–K).

### 3.8. Immune Cell Infiltration Analysis

In this study, through the analysis of immune cell infiltration, we systematically revealed the changes in the immune microenvironment of AF tissues and further explored the potential connections between autophagy and metabolic reprogramming-related molecules and immune cells.

**Figure 7:**
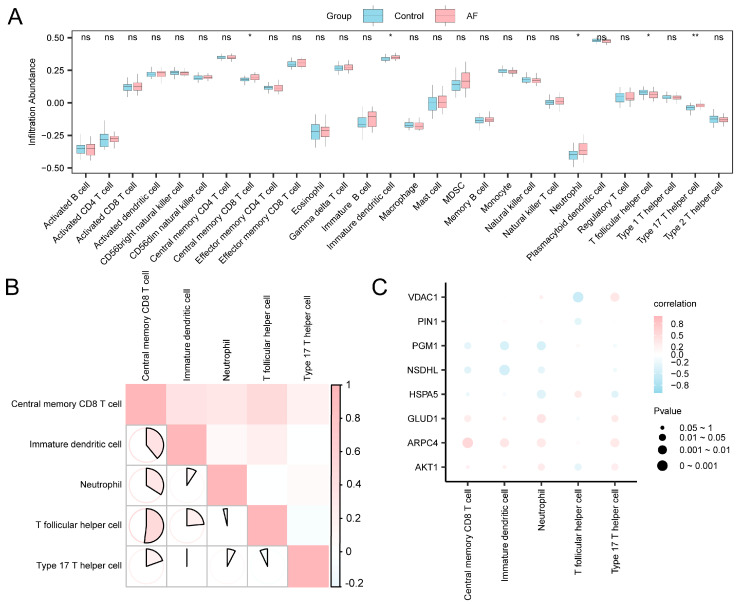
Immune Infiltration Analysis Using the ssGSEA Algorithm. (**A**) Comparison of immune cell infiltration between the control and AF groups in the combined GEO datasets. Blue represents the SR group and pink represents the AF group. (**B**) Heatmap showing correlations among immune cell infiltration levels. The depth of color reflects the strength of the correlation. The range of absolute values of correlation coefficients (r-values) illustrates the correlation of immune cells within each group. (**C**) Bubble plot illustrating the correlation between A&MRRDEGs and immune cell infiltration in the combined datasets. Statistical significance is indicated as follows: *p* ≥ 0.05 (ns, not significant); *p* < 0.05 (*); *p* < 0.01 (**). Pink bubbles indicate positive correlations and blue bubbles indicate negative correlations, with color intensity reflecting correlation strength. Abbreviations: AF, Atrial fibrillation; A&MRRDEGs, Autophagy- and metabolic reprogramming-related differentially expressed genes; GEO, Gene Expression Omnibus; ssGSEA, Single-sample gene set enrichment analysis.

The abundance of immune cell infiltration within the expression matrix of the combined datasets was determined for 28 distinct types of immune cells though ssGSEA. Comparative plots illustrating the variations in the infiltration abundance of reactive immune cells (Figure 7A) demonstrated that all five categories of them exhibited statistical significance (*p* < 0.05), specifically central memory CD8+ T cells, immature dendritic cells, neutrophils, T follicular helper cells (Tfh), and type 17 T helper cells.

Figure 7B correlates immune cell infiltration levels with the combined datasets. The results indicated a significant positive correlation in over half of the immune cell types, with the most pronounced correlation identified between central memory CD8+ T cells and T helper cells (r = 0.519, *p* < 0.05). The bubble plot (Figure 7C) illustrated strong correlations between A&MRRDEGs and most immune cell populations, highlighting their potential immunological relevance. Notably, *ARPC4* demonstrated the strongest positive correlation with central memory CD8 T+ cells (r = 0.565; *p* < 0.05).

### 3.9. Validation in the TAC Mouse Model

**Figure 8:**
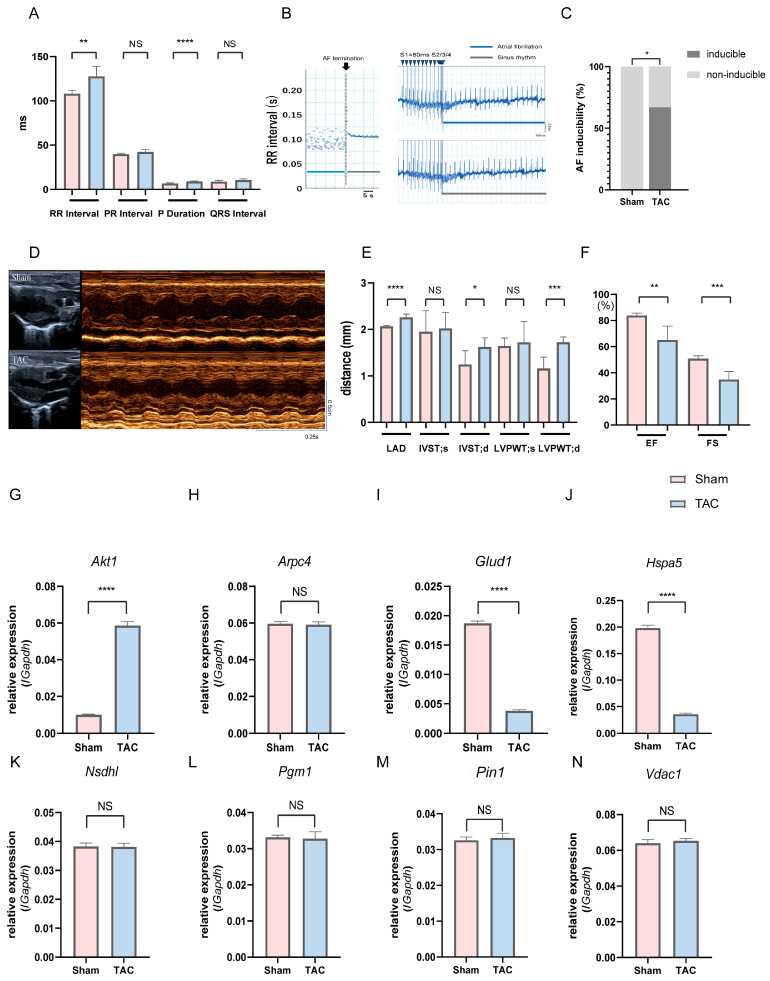
Electrophysiological assessment, AF inducibility, cardiac function, and relative mRNA expression of key genes in sham and TAC mice on postoperative day 14. (**A**) Surface ECG recordings in sham and TAC mice. (**B**) Left: RR interval–time plot during termination of induced AF; the dotted line indicates AF termination. Right: Representative ECG traces showing AF induction in a TAC mouse (top) but not in a sham mouse (bottom). Blue arrowheads indicate pacing stimuli. (**C**) AF inducibility rate in each group (*n* = 6), analyzed using Fisher’s exact test. (**D**) Representative M-mode echocardiographic images. (**E**) Echocardiographic measurements: LAD, IVST (d/s), LVPWT (d/s). (**F**) EF and FS. (**G**–**N**) Relative mRNA expression of key genes in atrial tissue: *Akt1* (**G**), *Arpc4* (**H**), *Glud1* (**I**), *Hspa5* (**J**), *Nsdhl* (**K**), *Pgm1* (**L**), *Pin1* (**M**), and *Vdac1* (**N**). Data are expressed as mean ± SD. *p* < 0.05 (*), *p* < 0.01 (**), *p* < 0.001 (***), *p* < 0.0001 (****) versus sham group; NS, Not significant. Abbreviations: AF, Atrial fibrillation; ECG, Electrocardiogram; LAD, Left atrial diameter; IVST, Interventricular septal thickness; LVPWT, Left ventricular posterior wall thickness; EF, Ejection fraction; FS, Fractional shortening; TAC, Transverse aortic constriction; UCG, Ultrasonic cardiography.

To validate the in vivo relevance of the identified key genes observed in human atrial tissue datasets, we further employed mice subjected to TAC, a well-established experimental model of AF [48,49]; we examined whether these gene expression changes could be recapitulated in an independent and controlled in vivo setting. Compared with sham controls, TAC mice exhibited significant prolongation of P-wave duration and RR interval on surface electrocardiography (ECG), and AF induction was observed in 67% (4/6) of TAC mice, whereas no AF episodes occurred in the sham group (*p* < 0.05, Figure 8A–C; Table S6). In the TAC group, mice with inducible AF showed no apparent differences in general condition or activity compared with mice without inducible AF, and no obvious body weight loss (23.6 ± 1.0 g vs. 23.6 ± 0.6 g). Echocardiography further demonstrated marked left atrial enlargement, increased left ventricular wall thickness, reduced EF, and FS in TAC mice, indicating atrial reprogramming accompanied by impaired cardiac function (Figure 8D–F; Table S7). Then, we assessed the atrial expression of the eight key genes via quantitative PCR (qPCR). *Glud1* and *Hspa5* expressions significantly reduced and *Akt1* expression significantly increased in TAC mice compared to those in sham controls (*p* < 0.0001, Figure 8G–J). Taken together, the eight key A&MRRDEGs identified through transcriptomic analyses of human atrial samples were validated in a TAC-AF murine model, supporting their relevance to AF pathogenesis.

## 4. Discussion

In this study, we performed transcriptomic analyses using GEO datasets comprising atrial samples from patients with AF and those with SR and further validated our findings in a murine AF model induced by TAC. Our analyses demonstrated that the expression profiles of the A&MRRGs were markedly altered in AF. Notably, across species and experimental models, *HSPA5 and AKT1* showed consistent changes in expression and were considered to be involved in AF pathogenesis. Within the scope of our investigation, no evidence was found to reveal that these genes might be potentially pivotal in vivo, linking autophagy and metabolic reprogramming to AF.

Although upstream therapies have been explored for the prevention and early treatment of AF, their success has been limited. For instance, treatments targeting the renin–angiotensin system, including angiotensin-converting enzyme inhibitors, have shown variable effectiveness in reducing the incidence of AF [50,51].

From the perspective of metabolic disorders, even strict glycemic control has not been effective in preventing the new onset of AF [52]. One possible explanation is that AF is a multifactorial disease, with diverse patient backgrounds and heterogeneous underlying mechanisms [3]. Autophagy has also been implicated in the development of AF, as supported by previous studies [53,54]. While the interplay between metabolic reprogramming and autophagy has been extensively investigated in cancer [55], it has not been explored in cardiovascular diseases, particularly AF. In the study, we identified the presence of A&MRRDEGs and their expression patterns in human atrial tissue. From a therapeutic perspective, because AF arises from multifactorial disease mechanisms, targeting common signaling pathways or molecules that operate across multiple pathogenic processes may provide an effective strategy. Our findings suggest that *HSPA5* and *AKT1*, identified in this study, may represent such candidates.

*HSPA5* [also known as glucose-regulated protein 78 (GRP78)] encodes a highly conserved member of the heat-shock protein 70 (HSP70) family that resides within the lumen of the endoplasmic reticulum (ER) [56]. It plays a crucial role in protein folding, assembly, and quality control, thereby maintaining ER homeostasis. In addition, HSPA5 serves as a crucial regulator of the unfolded protein response, facilitating cellular adaptation to ER stress and protecting against proteotoxic damage. In the present study, *HSPA5* expression decreased in atrial samples from patients with AF. In particular, *HSPA5* showed the strongest contribution to the diagnostic AF model and demonstrated the highest functional similarity to the other key genes. A previous study has also reported reduced HSPA5 protein levels in atrial tissue from AF patients [57], which is consistent with our results and indicates an association between *HSPA5* and AF. With regard to autophagy and metabolic reprogramming, *HSPA5* is known to be induced under metabolic stress conditions such as glucose deprivation, where it responds to enhanced ER stress and serves as a critical trigger of stress-induced autophagy [58]. Thus, *HSPA5* may act as a key gene linking metabolic reprogramming and autophagy. In this context, reduced *HSPA5* expression in AF could potentially lead to impaired autophagic mechanisms, thereby contributing to AF pathophysiology. However, the direct contribution of *HSPA5* to AF pathogenesis remains unclear and warrants further investigation.

AKT1, activated as a downstream effector of PI3K, is critical for cardiomyocyte survival, growth, and proliferation. Dysregulation of the PI3K/AKT pathway contributes to an array of cardiac disorders, such as myocardial infarction, heart failure, and cardiac hypertrophy [59]. In addition, the PI3K/AKT pathway is well established as a central regulator of metabolic processes, including glucose and lipid metabolism, as well as autophagy [60,61,62]. Dysregulation of this pathway is therefore thought to contribute to metabolic disturbances and impaired autophagic activity. However, the precise role of AKT in AF remains unclear and warrants further investigation. Although we did not examine AKT phosphorylation or protein expression levels in the present study, pathway analysis demonstrated enrichment of PI3K/AKT signaling-related genes, suggesting its potential involvement in AF pathophysiology.

Glutamate dehydrogenase 1 (GLUD1), encoded by the *GLUD1* gene, is a mitochondrial enzyme that plays a central role in glutamine/glutamate metabolism to tricarboxylic acid cycle anaplerosis and cellular energy/redox homeostasis [63]. In addition, GLUD1-related amino acid metabolism has been reported to influence mTORC1 signaling and thereby modulate autophagy [64]. In our study, *GLUD1* was upregulated in human atrial tissue from patients with AF, whereas it was downregulated in the murine TAC model. This discordant pattern precludes interpreting *GLUD1* as a conserved, universally AF-related molecular factor based on our current data alone. Accordingly, it remains unclear whether GLUD1 contributes to AF pathogenesis or instead represents a secondary consequence or a bystander change. Further studies are needed to clarify this issue.

In addition to *HSPA5* and *AKT1*, our analysis of human atrial datasets identified several other candidate genes that may serve as potential regulators in AF. Among them, *VDAC1* has been reported to be upregulated and may contribute to atrial fibrosis [65], whereas the relevance of the remaining candidates to AF has not yet been established. The human datasets used in the study were derived from patients with persistent or permanent AF, whereas the mouse model represents a predisposition to AF induction. Therefore, the alterations of gene expression in the remaining candidates may reflect the consequences of long-term persistence of AF. Further studies will be required to elucidate their potential roles in the pathogenesis of AF.

Moreover, we evaluated the diagnostic utility of these key genes for AF. Their expression levels demonstrated favorable diagnostic performance in DCA, ROC, and calibration analyses, both as an integrated model and at the level of individual genes. These findings suggest that even if these genes do not directly contribute to AF pathogenesis, they may still hold value as predictive biomarkers; thus, their validation in independent external cohorts is warranted. Although the acquisition of atrial tissue for gene expression analysis is quite challenging, samples obtained during open-heart surgery may offer particularly valuable insights for clinical and translational research. Given that AF often occurs paroxysmally and can lead to serious complications such as heart failure and stroke, the ability to predict its onset is of substantial clinical importance. Postoperative AF is also associated with adverse outcomes, raising the possibility that our findings may be applicable to intraoperative atrial tissue for AF risk prediction [57]. With the recent development of less invasive atrial biopsy techniques [66], gene expression profiling of atrial biopsy specimens may provide a novel approach for predicting AF in clinical practice.

In terms of the therapeutic potential of eight A&MRRDEGs identified in this study, the AKT inhibitor capivasertib is already available in clinical practice for the treatment of breast cancer [67]. However, an association between AF and AKT inhibitor therapy has not been reported so far, possibly because the clinical trials were not designed to evaluate AF risk, enrolled patients with advanced malignancy, and had relatively limited follow-up durations. In the future, as the number of patients treated with AKT inhibitors and the follow-up duration increase, an association between AKT and AF may be identified. Therefore, future observational studies in patients receiving AKT inhibitors may be warranted to assess AF risk.

The following limitations of this study warrant a mention. A primary limitation is the constrained number of atrial specimens; thus, future studies with more atrial samples and independent cohorts will be required. Second, it remains unclear whether the genes identified represent causal drivers of AF or consequences of the disease, and further investigations are needed to address this question. Third, our in vivo validation was restricted to a TAC murine model. Although TAC has been extensively studied and is widely used as an AF-relevant model, it primarily reflects pressure overload-driven remodeling and may not fully recapitulate the chronic and multifactorial nature of human AF. In addition, species-specific differences in cardiac electrophysiology between mice and humans warrant caution when interpreting and extrapolating these findings. Therefore, future studies using additional AF animal models may also be informative. Fourth, most findings were derived from mRNA expression data, and protein-level validation was not performed. Finally, although the diagnostic model and pathway analyses suggest potential clinical applications, these implications remain preliminary and require confirmation in independent cohorts as well as further mechanistic studies.

## 5. Conclusions

This study suggests that autophagy and metabolic reprogramming are involved in the pathogenesis of AF, with *AKT1* and *HSPA5* showing pivotal associations across human atrial samples and the TAC mouse model. While the precise roles of these genes in AF development remain to be clarified, these results offer a mechanistic understanding linking autophagy, metabolic reprogramming, and AF. These results may also offer potential clues for future diagnostic approaches, although further validation will be required.

## Data Availability

All data supporting the findings of this study are available within the paper and its Supplementary Information.

## Supplementary Materials

The following supporting information can be downloaded at: https://www.mdpi.com/article/10.3390/jcdd13020082/s1, Figure S1: Batch effect removal of GSE2240, GSE79768, and GSE14975; Figure S2: Gene set variation analysis (GSVA); Figure S3: Regulatory network of key genes. Table S1: GEO microarray chip information; Table S2: List of primers used for analysis of the expressions of key genes in mice by RT-qPCR; Table S3: Results of GO and KEGG enrichment analysis for A&MRRDEGs; Table S4: Results of GSEA for combined datasets; Table S5: Results of GSVA for combined datasets; Table S6: Measurements of body weight and surface ECGs in Sham (N = 6) and TAC (N = 6) mice on the 14th day after undergoing TAC or sham surgery; Table S7: Cardiac function assessment via echocardiograms between Sham (N = 6) and TAC (N = 6) mice on the 14th day after undergoing TAC or sham surgery. Datasheet S1: ARGs-7327; Datasheet S2: MRRGs-1424; Datasheet S3: AMRRGs-1137; Datasheet S4: Logoutput; Datasheet S5: mRNA-miRNA; Datasheet S6: mRNA-TF.

## Abbreviations

The following abbreviations are used in this manuscript:

AF	Atrial fibrillation
ARGs	Autophagy-related genes
AUC	Area under the curve
A&MRRGs	Autophagy- and metabolic reprogramming-related genes
A&MRRDEGs	Autophagy- and metabolic reprogramming-related differentially expressed genes
DEGs	Differentially expressed genes
EF	Ejection fraction
ERP	Effective refractory period
FDR	False discovery rate
FS	Fractional shortening
GEO	Gene Expression Omnibus
GO	Gene ontology
GSEA	Gene set enrichment analysis
GSVA	Gene set variation analysis
KEGG	Kyoto Encyclopedia of Genes and Genomes
LAD	Left atrial diameter
LASSO	Least absolute shrinkage and selection operator
LVEDD	Left ventricular end-diastolic diameter
LVESD	Left ventricular end-systolic diameter
LVPWT	Left ventricular posterior wall thickness
MRRGs	Metabolic reprogramming-related genes
PCA	Principal component analysis
RF	Random forest
ROC	Receiver operating characteristic
SR	Sinus rhythm
TAC	Transverse aortic constriction
TF	Transcription factors
