# Supplementary material for: Bioinformatics Analysis of Genes Associated with Autophagy and Metabolic Reprogramming in Atrial Fibrillation
Source: J Cardiovasc Dev Dis. 2026 Feb 8;13(2):82. doi: 10.3390/jcdd13020082 (PMC12942172; doi:10.3390/jcdd13020082)
Supplement: Supplementary file 1 [file jcdd-13-00082-s001.zip › Supplemetal_Figures.pdf]

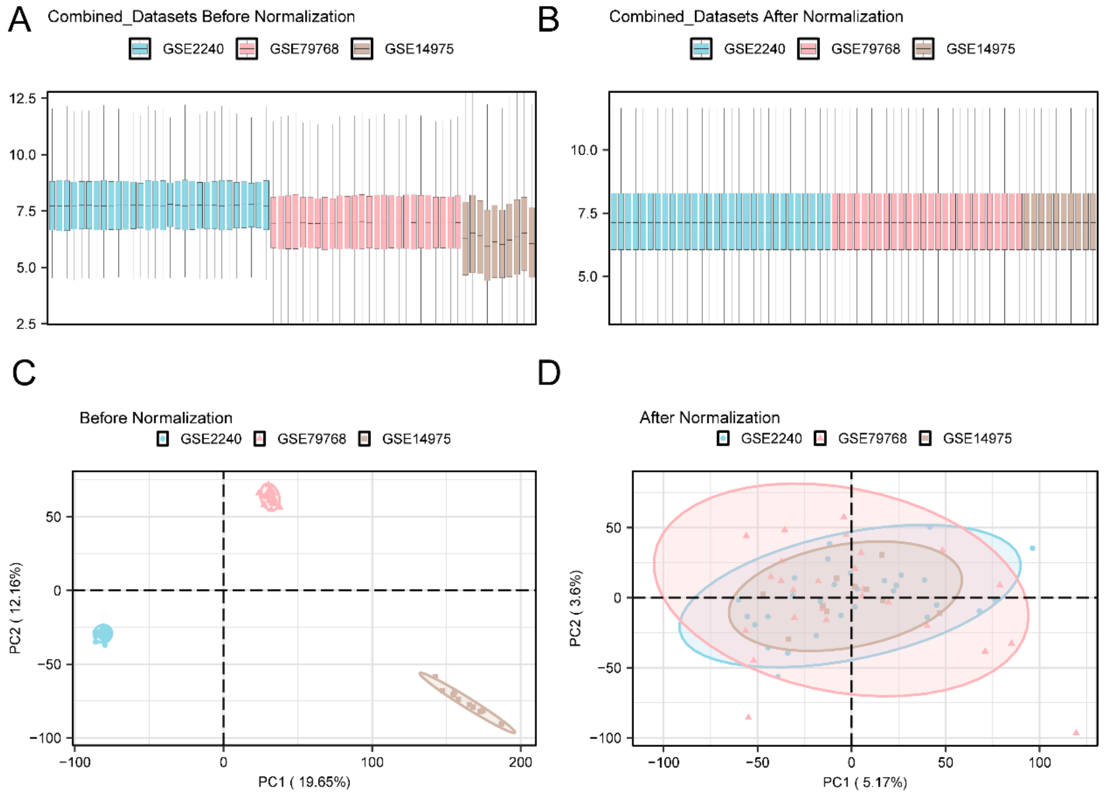

Figure S1. Batch effect removal of GSE2240, GSE79768, and GSE14975. (A) Distribution box plot of integrated GEO dataset (combined datasets) prior to batch processing. (B) Distribution boxplots of post-batch integrated GEO datasets (combined datasets). (C) PCA plot of the datasets before debatching. (D) PCA following batch processing of the integration of GEO dataset (combined datasets) of PCA. Abbreviations: AF, Atrial fibrillation; PCA, Principal component analysis. The atrial fibrillation (AF) dataset GSE2240 is blue, the AF dataset GSE79768 is pink, and the AF dataset GSE14975 is brown.

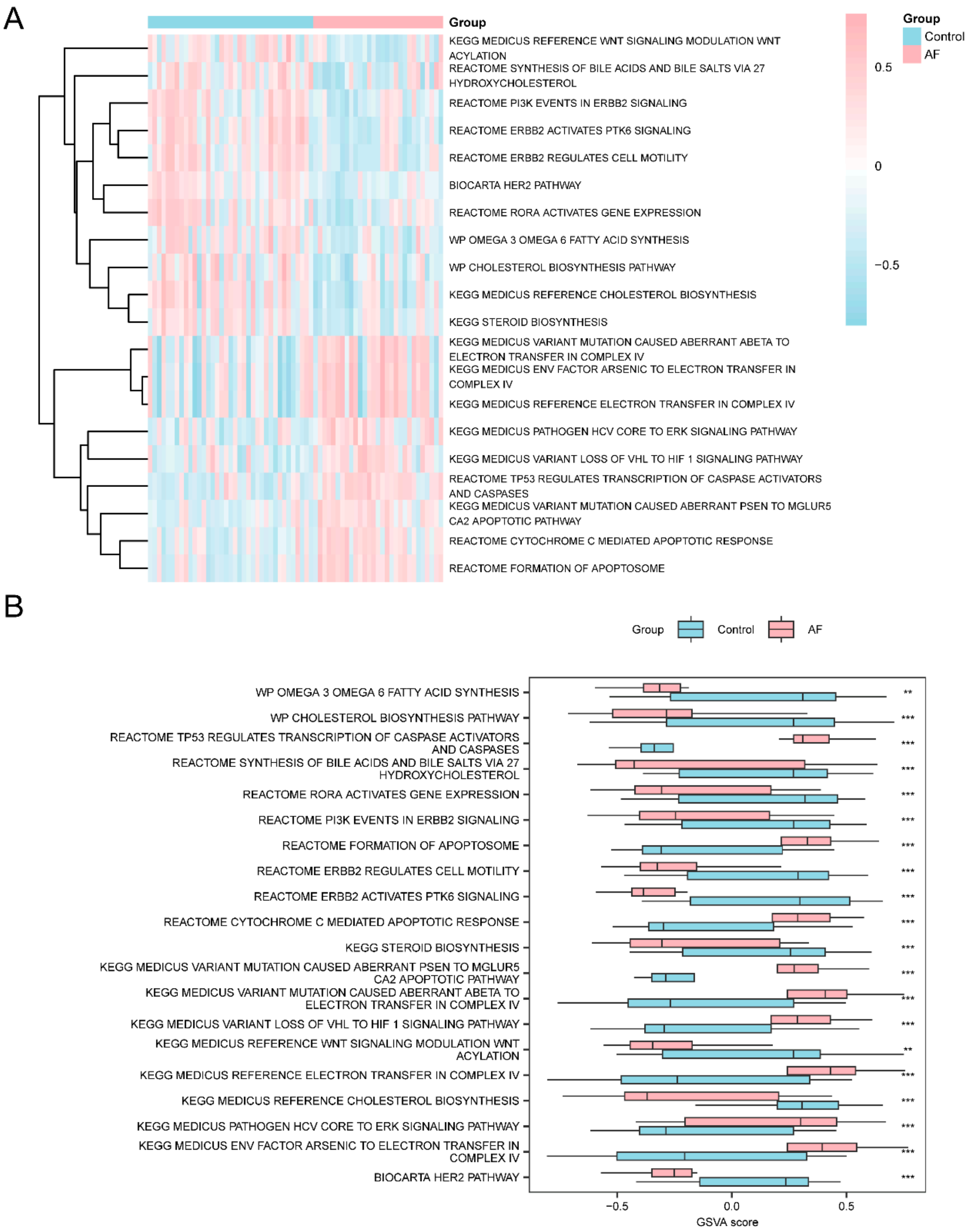

Figure S2. Gene set variation analysis (GSVA). (A–B) Heat map (A) and comparison (B) of GSVA results for atrial fibrillation (AF) and control groups in the combined GEO datasets. Abbreviations: AF, Atrial fibrillation; GSVA, Gene set variation analysis. ** represents p-value < 0.01, highly statistically significant; *** represents p-value < 0.001, highly statistically significant. Pink represents the atrial fibrillation (AF) group and blue represents the control group. GSVA set is

used to select p-values < 0.05, and p-values are correction using the Benjamini–Hochberg (BH) procedure. Blue represents low concentration in heatmap, pink represents high concentration.

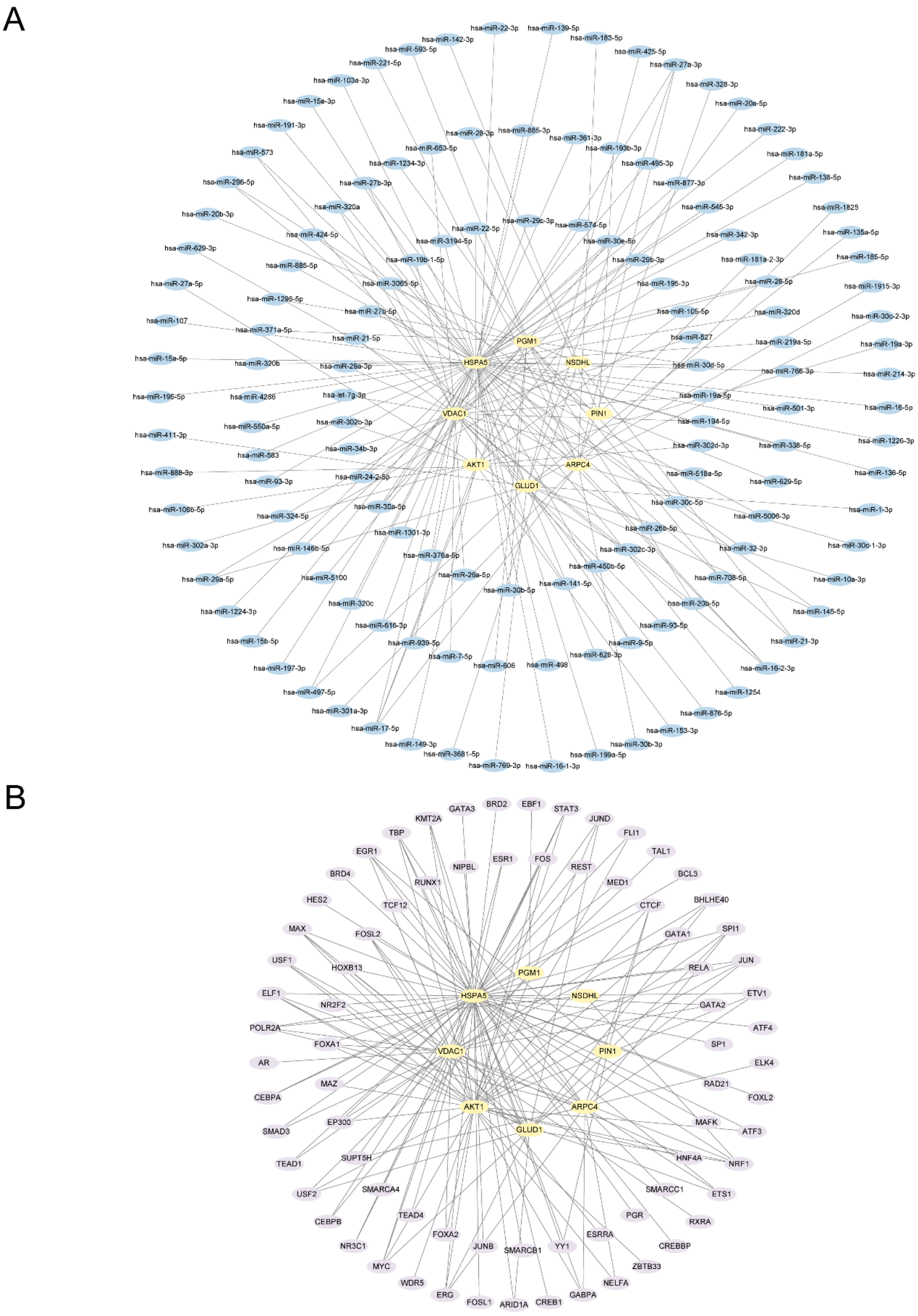

Figure S3. Regulatory network of key genes. (A) Autophagy- & metabolic reprogramming related differentially expressed genes (A&MRRDEGs); mRNA, the miRNA regulatory network. (B) mRNA-TF regulatory network of A&MRRDEGs. Abbreviations: A&MRRDEGs, Autophagy- & metabolic reprogramming-related differentially expressed genes; TF, Transcription factor. mRNAs are shown in yellow, miRNAs in blue, and TFs in purple.
