# Supplementary material for: Bioinformatics Analysis of Genes Associated with Autophagy and Metabolic Reprogramming in Atrial Fibrillation
Source: J Cardiovasc Dev Dis. 2026 Feb 8;13(2):82. doi: 10.3390/jcdd13020082 (PMC12942172; doi:10.3390/jcdd13020082)
Supplement: Supplementary file 1 [file jcdd-13-00082-s001.zip › Supplemtal Tables.pdf]

Table S1. GEO Microarray Chip Information

	GSE2240	GSE79768	GSE14975
Species	Homo sapiens	Homo sapiens	Homo sapiens
Platform	GPL96	GPL570	GPL570
Samples in AF group	10	14	5
Samples in Control group	20	12	5
PMID	PMID:15817885	PMID:27494721	PMID:20117462

GEO, Gene Expression Omnibus; AF, Atrial Fibrillation.

Table S2. List of primers used for analysis of the expressions of key genes in mice by RT-qPCR

Genes	Primers (5'-3')
Akt1	Forward: TAGGCATCCCTTCCTTACG<br>Reverse: CACAATCTCCGCACCATAG
Arpc4	Forward: TCGTGGAACGACACAACAAGCC<br>Reverse: CGGACAGAGTTGATGGAGCCTT
Glud1	Forward: TCCGTTACAGCACTGACGTGAG<br>Reverse: ACGCCTGCTTTAGCACCTCCAA
Hspa5	Forward: TGTCTTCTCAGCATCAAGCAAGG<br>Reverse: CCAACACTTCCTGGACAGGCTT
Nsdhl	Forward: TTAACCGCAGCCATTCGTCCTC<br>Reverse: GGTGAAGTCCACCAGGTTTTCC
Pgm1	Forward: CAACGCACTGAAGGAGCTACTC<br>Reverse: GGCACCAAGTTCTTCACAGAGG
Pin1	Forward: GAGGAAGACTTTGAATCTCTGGC<br>Reverse: TGTCCGTAGAGCAAACGACGCA
Vdac1	Forward: AGTGACCCAGAGCAACTTCGCA<br>Reverse: CAGGCGAGATTGACAGCAGTCT
GAPDH	Forward: TGTGATGGGTGTGAACCACGAGAA<br>Reverse: GAGCCCTTCCACAATGCCAAAGTT

Table S3. Results of GO and KEGG enrichment analysis for A&MRRDEGs.

Ontology	ID	Description	GeneRatio	BgRatio	pvalue	p.adjust
BP	GO:0006090	pyruvate metabolic process	22/263	106/18800	7.20476 e-20	3.04906 e-16
BP	GO:0006091	generation of precursor metabolites and energy	37/263	494/18800	6.59786 e-17	1.39611 e-13
BP	GO:0072521	purine-containing compound metabolic process	30/263	416/18800	1.90649 e-13	2.56312 e-10
BP	GO:0019693	ribose phosphate metabolic process	29/263	394/18800	2.9571 e-13	2.56312 e-10
BP	GO:0009150	purine ribonucleotide metabolic process	28/263	366/18800	3.02825 e-13	2.56312 e-10
CC	GO:0005759	mitochondrial matrix	34/264	473/19594	1.5423 e-15	6.72444 e-13
CC	GO:1990204	oxidoreductase complex	15/264	120/19594	7.88053 e-11	1.71796 e-08
CC	GO:0005925	focal adhesion	25/264	419/19594	5.60526 e-10	8.14631 e-08
CC	GO:0030055	cell-substrate junction	25/264	428/19594	8.69866 e-10	9.48154 e-08
CC	GO:0098798	mitochondrial protein-containing complex	19/264	281/19594	9.5969 e-09	8.3685 e-07
MF	GO:0045296	cadherin binding	24/264	333/18410	9.39133 e-11	3.69109 e-08
MF	GO:0051287	NAD binding	11/264	51/18410	1.22832 e-10	3.69109 e-08

Ontology	ID	Description	GeneRatio	BgRatio	pvalue	p.adjust
MF	GO:0140297	DNA-binding transcription factor binding	26/264	470/18410	4.48495 e-09	8.98484 e-07
MF	GO:0031625	ubiquitin protein ligase binding	20/264	298/18410	1.23178 e-08	1.85075 e-06
MF	GO:0044389	ubiquitin-like protein ligase binding	20/264	317/18410	3.46163 e-08	4.16087 e-06
KEGG	hsa01200	Carbon metabolism	23/210	115/8779	2.04777 e-15	5.73376 e-13
KEGG	hsa04066	HIF-1 signaling pathway	19/210	108/8779	7.0643 e-12	9.89002 e-10
KEGG	hsa01230	Biosynthesis of amino acids	16/210	75/8779	1.59872 e-11	1.49214 e-09
KEGG	hsa05230	Central carbon metabolism in cancer	14/210	70/8779	7.72771 e-10	5.4094 e-08
KEGG	hsa00010	Glycolysis / Gluconeogenesis	13/210	67/8779	4.71206 e-09	2.63875 e-07

GO: Gene ontology. BP: Biological process. CC: Cellular component. MF: Molecular function. KEGG: Kyoto Encyclopedia of Genes and Genomes. A&MRRDEGs: Autophagy- & metabolic reprogramming-related differentially expressed genes.

Table S4. Results of GSEA for combined datasets.

ID	setSize	enrichmentScore	NES	pvalue	p.adjust	qvalue
FOROUTAN_PRODRANK_TGFB_EMT_DN	67	0.466305719	1.629776008	0.00569407	0.037081055	0.028377896
SCHOEN_NFKB_SIGNALING	29	0.651496435	1.820765447	0.002315473	0.018772108	0.014366175
KEGG_JAK_STAT_SIGNALING_PATHWAY	119	0.584703297	2.040041801	3.11 e-07	9.10 e-06	6.97 e-06
PID_PI3KCI_PATHWAY	39	0.717527035	2.144884993	3.19 e-05	0.000505528	0.000386878
MARTINEZ_TP53_TARGETS_UP	465	0.346383408	1.393974432	0.002836042	0.022125697	0.016932655
FLECHNER_BIOPSY_KIDNEY_TRANSPLANT_REJECTED_VS_OK_UP	63	0.826101818	2.671315115	1.00 e-10	7.66 e-09	5.86 e-09
NAKAYAMA_SOFT_TISSUE_TUMORS_PCA1_UP	56	0.83644214	2.640978682	1.00 e-10	7.66 e-09	5.86 e-09
SCHUETZ_BREAST_CANCER_DUCTAL_INVASIVE_UP	284	0.672442386	2.593159046	1.00 e-10	7.66 e-09	5.86 e-09
WALLACE_PROSTATE_CANCER_RACE_UP	212	0.688753041	2.574018496	1.00 e-10	7.66 e-09	5.86 e-09
VERHAAK_GLIOBLASTOMA_MESENCHYMAL	180	0.701738531	2.569502589	1.00 e-10	7.66 e-09	5.86 e-09

GSEA: Gene Set Enrichment Analysis.

Table S5. Results of GSVA for combined datasets.

	logFC	AveExpr	t	P.Value
REACTOME ERBB2 ACTIVATES PTK6 SIGNALING	0.750636068	0.01086235	5.890490263	7.80 e-08
REACTOME TP53 REGULATES TRANSCRIPTION OF CASPASE ACTIVATORS AND CASPASES	0.726526855	0.004061289	5.974465557	5.45 e-08
KEGG MEDICUS REFERENCE CHOLESTEROL BIOSYNTHESIS	0.689662063	0.017964346	4.868146663	5.25 e-06
KEGG MEDICUS REFERENCE ELECTRON TRANSFER IN COMPLEX IV	0.651573266	0.041580023	3.787178767	0.000286121
KEGG MEDICUS ENV FACTOR ARSENIC TO ELECTRON TRANSFER IN COMPLEX IV	0.650571877	0.037725514	3.879526586	0.000207958
KEGG MEDICUS VARIANT MUTATION CAUSED ABERRANT ABETA TO ELECTRON TRANSFER IN COMPLEX IV	0.631867684	0.031289104	3.891428116	0.000199514
REACTOME FORMATION OF APOPTOSOME	0.626103067	0.013119124	4.997381686	3.15 e-06
REACTOME ERBB2 REGULATES CELL MOTILITY	0.616523778	0.018997104	4.779340039	7.44 e-06
WP CHOLESTEROL BIOSYNTHESIS PATHWAY	0.580512444	0.025227647	3.95830351	0.000157836
REACTOME RORA ACTIVATES GENE EXPRESSION	0.576747975	0.013675565	4.261902125	5.29 e-05
BIOCARTA HER2 PATHWAY	0.571531896	0.011609163	5.31528733	8.70 e-07
REACTOME CYTOCHROME C MEDIATED APOPTOTIC RESPONSE	0.568855507	0.005146261	4.655627153	1.20 e-05
KEGG MEDICUS VARIANT LOSS OF VHL TO HIF 1 SIGNALING PATHWAY	0.559871099	0.007264345	4.240192413	5.73 e-05

REACTOME PI3K EVENTS IN ERBB2 SIGNALING	0.554995395	0.01561174	4.271219085	5.12 e-05
KEGG STEROID BIOSYNTHESIS	0.554059886	0.001874644	4.359630911	3.69 e-05
WP OMEGA 3 OMEGA 6 FATTY ACID SYNTHESIS	0.549438964	0.012078926	3.975579646	0.000148508
KEGG MEDICUS PATHOGEN HCV CORE TO ERK SIGNALING PATHWAY	0.547707211	0.002345928	4.066143901	0.000107639
REACTOME SYNTHESIS OF BILE ACIDS AND BILE SALTS VIA 27 HYDROXYCHOLESTEROL	0.547441221	0.000751149	3.700610873	0.000384254
KEGG MEDICUS VARIANT MUTATION CAUSED ABERRANT PSEN TO MGLUR5 CA2 APOPTOTIC PATHWAY	0.543566425	0.007520797	4.758254831	8.08 e-06
KEGG MEDICUS REFERENCE WNT SIGNALING MODULATION WNT ACYLATION	0.531453537	0.038854188	3.688112288	0.000400829

GSVA: Gene Set Variation Analysis.

Table S6. Measurements of body weight and surface ECGs in Sham (N=6) and TAC (N=6) mice on the 14th day after undergoing TAC or sham surgery.

Avg.	Sham	TAC	P value
Body Weight (g)	23.71±0.7559	23.60±1.001	0.8293
Heart Rate (BPM)	586.3±21.3	521±19.3	0.0002***
RR Interval (ms)	108.1±3.668	127.7±11.31	0.0163*
PR Interval (ms)	39.93±1.057	42.26±2.975	0.1000
P Duration (ms)	6.827±0.6360	9.013±0.5108	<0.0001****
QRS Interval (ms)	8.825±1.483	10.57±1.407	0.0624

Data are Mean ± SEM. N=6, Sham; N=6,TAC. SBP, Systolic Blood Pressure. DBP, Diastolic Blood Pressure. BPM, beat per minute. ****P < 0.0001 vs sham, ***P < 0.001 vs sham, **P < 0.01 vs sham, *P < 0.05 vs sham among the 14th underwent TAC or sham surgery.

Table S7. Cardiac function assessment via echocardiograms between Sham (N=6) and TAC (N=6) mice on the 14th day after undergoing TAC or sham surgery.

Avg	Sham	TAC	P value (Sham vs TAC)
LAD (ms)	2.067±0.02251	2.260±0.06812	<0.0001****
IVST; s (ms)	1.948±0.4518	2.020±0.3442	0.7636
IVST; d (ms)	1.248±0.2914	1.622±0.1979	0.0267*
LVPWT; s (ms)	1.642±0.1694	1.722±0.4440	0.6888
LVPWT; d (ms)	1.162±0.2398	1.725±0.1108	0.0004***
LVEDD (ms)	2.410±0.2535	2.153±0.4756	0.2704
LVESD (ms)	1.200±0.1532	1.415±0.3988	0.2459
EF%	83.83±1.820	65.00±10.72	0.0017**
FS%	50.79±2.049	34.87±6.156	0.0001***

Data are Mean ± SEM. N=6, Sham; N=6, TAC. LAD; left atrial diameter. IVST, d/s; systolic and diastolic interventricular septal thickness.LVPWT, d/s; systolic and diastolic left ventricular posterior wall thickness, LVEDD; leftventricular end-diastolic diameter. LVESD; left ventricularend-systolic diameter.EF%, Ejection fraction; FS%, Fractional shortening. ****P < 0.0001 vs sham, ***P < 0.001 vs sham, **P < 0.01 vs sham, *P < 0.05 vs sham among the 14th underwent TAC or sham surgery.
